# Supplementary material for: Mechanisms governing the pioneering and redistribution capabilities of the non-classical pioneer PU.1
Source: Nat Commun. 2020 Jan 21;11:402. doi: 10.1038/s41467-019-13960-2 (PMC6972792; doi:10.1038/s41467-019-13960-2)
Supplement: Supplementary file 7 — Source data [file 41467_2019_13960_MOESM7_ESM.zip › Source_Data/FigureS2/FigureS2B_MotifScanOutput/KmeansCluster9/homerResults/motif16.similar.html]

motif16

## Information for motif16

C
T
A
G
C
T
G
A
C
T
A
G
C
T
A
G
G
T
C
A
C
T
G
A
A
T
C
G
C
T
G
A
C
T
A
G
A
C
T
G
T
C
G
A
  
Reverse Opposite:  

A
C
G
T
T
G
A
C
A
G
T
C
A
G
C
T
A
T
G
C
G
A
C
T
A
C
G
T
G
A
T
C
A
G
T
C
A
G
C
T
A
G
T
C
  

|  |  |
| --- | --- |
| p-value: | 1e-24 |
| log p-value: | -5.752e+01 |
| Information Content per bp: | 1.865 |
| Number of Target Sequences with motif | 223.0 |
| Percentage of Target Sequences with motif | 9.80% |
| Number of Background Sequences with motif | 2048.1 |
| Percentage of Background Sequences with motif | 4.44% |
| Average Position of motif in Targets | 102.5 +/- 42.4bp |
| Average Position of motif in Background | 107.7 +/- 77.5bp |
| Strand Bias (log2 ratio + to - strand density) | -0.3 |
| Multiplicity (# of sites on avg that occur together) | 1.22 |
| Motif File: | file (matrix) reverse opposite |

### Similar de novo motifs found

|  |  |  |  |  |  |  |  |
| --- | --- | --- | --- | --- | --- | --- | --- |
| Rank | Match Score | Redundant Motif | P-value | log P-value | % of Targets | % of Background | Motif file |
| 1 | 0.938 | C T G A T C A G C T G A C T A G C T A G C G T A C T G A T A C G C T G A C T A G A C T G C G T A C T A G | 1e-21 | -50.119269 | 3.95% | 1.10% | motif file (matrix) |
| 2 | 0.791 | C T A G C A T G T C G A C T G A T A C G T C G A T C A G T C A G C G T A C T A G C T A G C G T A C T A G T C A G | 1e-15 | -36.345521 | 4.61% | 1.79% | motif file (matrix) |
